# Polyphenol-Rich Extract of *Apocynum venetum* L. Leaves Protects Human Retinal Pigment Epithelial Cells against High Glucose-Induced Damage through Polyol Pathway and Autophagy

**DOI:** 10.3390/nu16172944

**Published:** 2024-09-02

**Authors:** Jun Peng, Rahima Abdulla, Xiaoyan Liu, Fei He, Xuelei Xin, Haji Akber Aisa

**Affiliations:** 1 The State Key Laboratory Basis Xinjiang Indigenous Medicinal Plant Resource, Xinjiang Technical Institute of Physics and Chemistry, Chinese Academy of Sciences, Urumqi 830011, China; pengjunlhs@163.com (J.P.); rahima@ms.xjb.ac.cn (R.A.); liuxiaoyan_shawn@163.com (X.L.); hefei@ms.xjb.ac.cn (F.H.); 2 University of Chinese Academy of Sciences, Beijing 100039, China

**Keywords:** dietary polyphenols, *Apocynum venetum* L., diabetic retinopathy, polyol pathway, autophagy

## Abstract

Diabetic retinopathy (DR) is a specific microvascular problem of diabetes, which is mainly caused by hyperglycemia and may lead to rapid vision loss. Dietary polyphenols have been reported to decrease the risk of DR. *Apocynum venetum* L. leaves are rich in polyphenolic compounds and are popular worldwide for their health benefits as a national tea drink. Building on previous findings of antioxidant activity and aldose reductase inhibition of *A. venetum*, this study investigated the chemical composition of polyphenol-rich extract of *A. venetum* leaves (AVL) and its protective mechanism on ARPE-19 cells in hyperglycemia. Ninety-three compounds were identified from AVL by LC-MS/MS, including sixty-eight flavonoids, twenty-one organic acids, and four coumarins. AVL regulated the polyol pathway by decreasing the expression of aldose reductase and the content of sorbitol, enhancing the Na^+^K^+^-ATPase activity, and weakening intracellular oxidative stress effectively; it also could regulate the expression of autophagy-related proteins via the AMPK/mTOR/ULK1 signaling pathway to maintain intracellular homeostasis. AVL could restore the polyol pathway, inhibit oxidative stress, and maintain intracellular autophagy to protect cellular morphology and improve DR. The study reveals the phytochemical composition and protective mechanisms of AVL against DR, which could be developed as a functional food and/or candidate pharmaceutical, aiming for retina protection in diabetic retinopathy.

## 1. Introduction

Dietary polyphenols are natural phytochemical compounds, mainly from herbs, tea, and other beverages, which exhibit multiple biological properties, including antioxidant, antimicrobial, antiviral, anticancer, antiinflammatory, and antidiabetic activities [1]. Recently, the epidemiological survey between dietary polyphenol intake and type 2 diabetes shows that the polyphenol intake in diabetic people is lower than in non-diabetic people [2]. Researchers have also realized that dietary polyphenols can prevent and cure diabetes mellitus and its complications [3]. Diabetic retinopathy (DR) is a specific microvascular problem of diabetes and is mainly caused by hyperglycemia [4]. It is the leading cause of vision loss in adults of working age, and the population of DR will reach over 50 million by 2045 [5]. Prevention and early intervention are becoming increasingly important due to irreversibility in the late stage of DR. Polyphenol-rich products could treat type 2 diabetes mellitus and its complications by regulating metabolic disorders and alleviating stress pathways [6,7]. Hence, the utilization of dietary polyphenols for the prevention and treatment of DR holds significant value.

Diverse factors drive abnormal regulation of biochemical pathways during the development of DR, including the polyol pathway, glycation, myoinositol, oxidative stress, autophagy, and protein kinase C (PKC) [8]. Hyperglycemia-induced polyol pathway hyperactivity is considered to be one possible mechanism underlying the development of DR [9]. Aldose reductase (AR) is the first rate-limiting enzyme in the polyol pathway. Its over-expression causes the accumulation of sorbitol, resulting in edema and osmotic pressure damage and a decrease of Na^+^K^+^-ATPase activity, which aggravates diabetic retinopathy. Autophagy is an evolutionarily conserved lysosomal degradation involved in the development of DR. The molecular mechanism of autophagy is very complex, involving various autophagy-related proteins (ATG) with the AMPK/mTOR signaling pathway, as well as and the switch of anabolic and catabolic processes [6]. Polyphenols play a vital role in interfering with the polyol pathway and autophagy [6,10].

*Apocynum venetum* L., a perennial shrub plant of the Apocynaceae family, is widely distributed in Central Asia, North America, and the Mediterranean Coast [11]. Its leaves have a long history as a health tea or traditional Chinese medicine (TCM) for antihypertension in China [12] and are rich in multiple bioactive components such as phenolic acids, flavonoids, and polysaccharides. Studies on the bioactivities of *A. venetum* leaves revealed its antihypertension, antioxidation, antiaging, hepatoprotective, protecting nerve, and anticancer activity [13,14,15,16]. Polysaccharide extracts from *A. venetum* leaves significantly decreased the levels of fasting blood glucose, serum insulin, glycated serum protein, as well as serum lipid profiles; polysaccharide products also increased glycogen contents in liver and improved the oxidative damage in diabetic mice significantly [17]. Therefore, *A. venetum* leaves have potential hypoglycaemic effects. Our previous study found that total polyphenol components from *A. venetum* leaves (AVL) have good antioxidant activity [18] and inhibit aldose reductase in vitro [19], which indicated that AVL might prevent and intervene early in the development of DR.

This work aims to study the chemical components and the protective effect and possible mechanism of *A. venetum* leaves for human retinal pigment epithelial cells (ARPE-19) induced by high-glucose conditions. This study provides a basis for further research and application of *A. venetum* leaves to be used as a potential functional food and candidate pharmaceutical to treat DR.

## 2. Methods

### 2.1. Material and Reagents

*A. venetum* leaves were deposited at Xinjiang Technical Institute of Physics and Chemistry, Chinese Academy of Sciences, where they were identified and numbered (WY02685) by Associate Professor Chunfang Lu. They were collected from Altay region of Xinjiang in China in August of 2018.

Formic acid and acetonitrile for HPLC were obtained from Merck (Darmstadt, Germany). DL-glyceraldehyde, 3-(4,5-dimethylthiazol-2-yl)-2,5- diphenyl tetrazolium bromide (MTT), Dimethyl Sulfoxide (DMSO), D-glucose (all from Sigma, St. Louis, MO, USA). Epalrestat (Yuanye Bio-Technology, Shanghai, China) and Difrarel (Laboratoires Leurquin Mediolanum, Fontenay-sous-Bois, France). Aldose reductase was expressed in the laboratory at the Xinjiang Technology Institute of Physics and Chemistry. Human retinal pigment epithelium cell line (ARPE-19) was purchased from the Type Culture Collection of the Chinese Academy of Sciences (Shanghai, China). Dulbecco’s Modified Eagle Medium/Nutrient Mixture F-12 (DMEM/F-12), fetal bovine serum, streptomycin, and penicillin (all from Gibco, Carlsbad, CA, USA). Super RIPA Lysis Buffer (Beyotime Institute of Biotechnology, Shanghai, China).

### 2.2. Preparation of A. venetum Leaves Polyphenol Extraction (AVL)

The dried *A*. *venetum* leaves were extracted with 50% ethanol for 2 h with heat reflux extraction at 70 °C [18,20]. After two rounds of extraction, the polyphenol components of *A*. *venetum* leaves (AVL) were enriched by HPD-300 microporous resin (Cangzhou Bon Adsorber Technology Co., Cangzhou, China). The optimum conditions of purification were as follows: sample concentration was 6.0 mg/mL, flow rate was 2 BV/h, and maximum solution treatment capacity was 8 BV; The eluting agent was 5 BV purified water, with the total polyphenols being desorbed by 3 BV of 50% alcohol at the flow rate 2 BV/h [18,20].

### 2.3. Characterization of A. venetum Polyphenol Extraction (AVL)

#### 2.3.1. Qualitative Analysis

Qualitative analysis was conducted with Ultra-high Performance Liquid Chromatography coupled with hybrid quadrupole-Orbitrap high-resolution mass spectrometry (UHPLC-Q-Orbitrap-HRMS) (Thermo Fisher Scientific, Bremen, Germany). 

Ultra-high-performance liquid chromatography (UHPLC) analysis was performed with Dionex Ultimate 3000 RSLC system (Thermo Fisher Scientific, Waltham, MA, USA) with the diode array detector (DAD). Chromatographic separation was performed on the SHIMADZU Shim-pack GIS C18 column (4.6 × 250 mm, 5 μm) with mobile phases of solvent A (0.1% HCOOH) and solvent B (ACN) under 254 nm UV at the flow rate of 1.0 mL/min. The gradient is as follows: 0–24 min, 7–15% B; 24–39 min, 15–22% B; 39–54 min, 22–28% B; 54–57 min, 28–30% B; 57–62 min, 30–32% B; and 62–67 min, 32–50% B.

Mass spectrometry detection using Q Exactive mass spectrometer equipped with electrospray ion (ESI) source. The operating parameters for the MS condition were as positive and negative ESI spray voltage, 3.2 kV and −2.8 kV, respectively; sheath gas, 40 arb; auxiliary gas, 10 arb; curtain gas, 35; the heat temperature, 350 °C; the Q-Orbitrap collection range, 100–1500 *m*/*z*; the fragment ion scanning range, 50–1500 *m*/*z*; MS Resolution, 70,000 FWHM (*m*/*z* 200); MS^2^ Resolution, 17,500 FWHM (*m*/*z* 200). Xcalibur 4.0 software (Thermo Fisher Scientific, Waltham, MA, USA) was used to analyze data.

#### 2.3.2. Total Phenols Content (TPC)

The total phenol content test was performed according to the Folin–Ciocalteu colorimetric method with slight modification. Folin–Ciocalteu reagent was diluted 10 times before use. Briefly, sample solution (10 µL) and Folin–Ciocalteu reagent diluent (30 µL) were added to each well of a 96-well plate to incubate together for 2 min. Subsequently, 7.5% Na_2_CO_3_ (30 µL) and ddH_2_O (180 µL) were added to each well. The absorbance was measured at the wavelength λ = 765 nm. Additionally, the sample was replaced 50% aqueous methanol in the blank group. The total phenol content was calculated from a standard curve of gallic acid at concentrations.

### 2.4. Aldose Reductase Activity Assay

The inhibition of aldose reductase and the half inhibition concentration (IC_50_) were calculated according to the reference [21]. Briefly, Sample solution (4 µL), PBS buffer (146 µL), and aldose reductase solvent (10 µL) were successively added to 96-well plate. Then, nicotinamide adenine dinucleotide phosphate (NADPH) and DL-glyceraldehyde were mixed as a substrate, which was added to incubate for 30 min. The absorption was read at 340 nm. As for blank, 2 μL samples were replaced by DMSO. Quercetin was used as the positive control.

### 2.5. Cell Culture and High Glucose Damage Model

ARPE-19 cells were cultured in the complete growth medium containing DMEM/F-12 with 10% fetal bovine serum, 100 μg/mL streptomycin, and 100 U/mL penicillin. Cells were used between the fifth and fifteenth passages. For the high glucose damage model, ARPE-19 cells were exposed to 30 mM D-glucose and incubated at 37 °C in a humidified chamber of 5% CO_2_ for 48 h [22]. Untreated cells were cultured with 4.5 mM D-glucose as the normal group. 

For the treatment groups, after ARPE-19 cells (5 × 10^5^ cells) were cultured in medium with 30 mM glucose for 24 h, then they were treated with AVL (6.25, 12.5, and 25 μg/mL) under the high-glucose condition (HG, 30 mM D-glucose) for another 24 h in a 6-well plate. Positive groups with treatment of epalrestat (0.1 μM) and Difrarel (25 μg/mL) in the same condition [23,24,25].

### 2.6. Cell Viability Assay

MTT assay was carried out according to the previous description [26]. After ARPE-19 cells were cultured in medium with 30 mM glucose for 24 h, and then they were treated with AVL (6.25, 12.5, 25, 50, and 100 μg/mL) under the high-glucose condition (HG, 30 mM D-glucose) for another 24 h. The absorbance value was measured at a wavelength of 490 nm.

### 2.7. Evaluation of Polyol Pathway on Aldose Reductase mRNA Expression, Sorbitol Content, and Na^+^ K^+^-ATPase Activity

#### 2.7.1. Aldose Reductase mRNA Expression

AR mRNA expression was quantified in real-time quantitative PCR experiments with the SYBR green primer using the 7300 HT real-time PCR system (Applied Biosystems, Foster, CA, USA) [26]. Primer (BGI, Shenzhen, China) sequences used were as follows: 

AR Forward: 5-TTTTCCCATTGGATGAGTCGG-3 and Reverse: 5-ACGTGTCCAGAATGTTGGTGT-3; 

GADPH Forward: 5-GTCTCCTCTGACTTCAACAGCG-3 and Reverse: 5-ACCACCCTGTTGCTGTAGCCAA-3.

#### 2.7.2. Na^+^ K^+^-ATPase Activity Measurements

The production of Na^+^K^+^-ATPase activity was measured using the Na^+^ K^+^-ATPase Activity Detection Kit (Beijing Solarbio Science & Technology Co., Ltd., Beijing, China) following the manufacturer’s instructions.

#### 2.7.3. The Content of Sorbitol Assay

The ARPE-19 Cells were boiled in tri-distilled water for 10 min, and the supernatants were obtained by centrifugation for 10 min, 8000× *g*, room temperature. Sorbitol content detection with supernatants was performed using a D-Sorbitol/Xylitol Assay Kit (Megazyme International Ireland Co., Ltd., Wicklow, Ireland) and protocol.

### 2.8. Evaluation of Glutathione and ROS

#### 2.8.1. The Content of the Reduced Glutathione (GSH)

The level of reduced glutathione was measured using Reduced Glutathione Assay Kit (Nanjing Jiancheng Bioengineering Institute, Nanjing, China) according to the direction [17]. 

#### 2.8.2. Examination of Intracellular ROS Generation

The intracellular ROS level in ARPE-19 cells with AVL was measured by fluorescence microscope with 2, 7-dichlorofluorescein diacetate (DCFH-DA) (Nanjing Jiancheng Bioengineering Institute, Nanjing, China). The treated cells were evaluated by fluorescence microscopy and microplate spectrophotometer according to the direction [22].

### 2.9. Cellular Morphology Electron Microscopy

The cellular morphology was detected by electron microscopy. Cells were fixed with 2.5% glutaraldehyde for 1 h at room temperature. Samples were fixed with 1% osmium tetroxide, dehydrated by successive acetone washes (50%, 70%, 90%, 100%) 4 and embedded with epoxy resin. After polymerization, ultrathin sections were cut using an ultramicrotome and stained with 2% uranyl acetate and Reynold’s lead citrate. Sections were assessed using a JEOL 1011 transmission electron microscope (JEOL, Tokyo, Japan) equipped with a GATAN Erlangshen CCD camera (Gatan, Pleasanton, CA, USA).

### 2.10. Western Blot

ARPE-19 cells were treated and collected by super RIPA lysis buffer; protein concentration was determined using Pierce BCA Protein Assay Kit (Thermo Scientific, Waltham, MA, USA). The method of western blots is performed according to the reference [26]. Equal amounts of protein samples were subjected to 6%, 8%, and 15% SDS-polyacrylamide gel electrophoresis before being transferred to a polypropylene fluoride (PVDF) membrane. The membrane was probed with antibodies against LC3A/B-II/I, P62, Beclin-1, ATG3, ATG5, ATG7, ATG12, ATG16L1, p-AMPK (Thr172), AMPK, p-mTOR, mTOR, p-ULK1(ser555), ULK1, GADPH, *β*-actin (all purchased from CST, Boston, MA, USA), and antialdose reductase (Abcam, Cambridge, MA, USA), respectively. The protein signal was amplified and observed with a fluorescent-labeled second antibody. The membranes were performed with an ECL Western Blot Detection System.

### 2.11. Statistical Analysis

The experiment results are presented as the mean ± standard deviation (SD). All statistical analyses were performed using GraphPad Prism 7.0 (GraphPad Software Inc., Boston, MA, USA). *p*-values were analyzed by one-way analysis of variance, which is a method of testing differences between more than two groups or treatments. The statistically significant result was represented as # *p* < 0.05, ## *p* < 0.01 versus NG, * *p* < 0.05, ** *p* < 0.01 versus HG.

## 3. Results and Discussion

### 3.1. The Chemical Composition 

**Figure 1:**
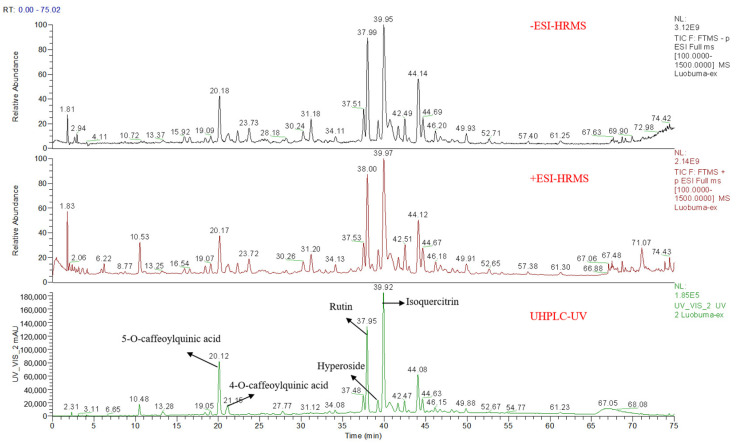
Total ion chromatograms and UHPLC chromatogram of AVL.

In this study, the total polyphenol content of AVL was 48.47 ±  0.41%, the IC_50_ value of AVL on AR was 17.38 ± 1.10 μg/mL, and the IC_50_ value of quercetin was 5.42 ± 0.40 μg/mL. Based on retention time (t_R_), molecular mass, fragment ions, standards, and previous references, 93 compounds were identified from AVL. The results are shown in Table 1, and the base peak chromatograms on positive and negative ion modes are shown in Figure 1. The polyphenolic composition of AVL included sixty-eight flavonoids, twenty-one organic acids, and four coumarins. Based on previous studies, the extract was rich in flavonoid-type phenolic compounds, which had a common C6-C3-C6 backbone structure. The flavonoids of AVL were the main flavonol and flavane derivatives, including glycosides of quercetin, kaempferol, and myricetin, as well as catechin compounds. The hydroxyl groups on the A-ring and B-ring of flavonoids can enhance the inhibition of AR, and the hydroxyl groups on C-5 and C-7 of the A-ring and C-3′ and C-4′ of the B-ring can significantly increase the inhibition on AR, while the hydrogenation of C2=C3 and the glycosylation on 7-OH and 4′-OH of flavonoids could reduce this inhibition [27]. The hydroxyl group of coumarin at C-6 and C-7 had an inhibitory effect on AR [28]. AR is the first rate-limiting enzyme involved in the polyol pathway; AR and the polyol pathway were considered as significant mechanisms to explain how hyperglycemia initiates diabetic retinopathy [29]. The dietary polyphenols are effective and safe components in functional foods for the treatment of chronic eye diseases [30]. It was reported that anthocyanins (such as cyanidin 3-O-galactosides), flavonols (such as quercetin and myricetin), and organic acids (such as neochlorogenic acid) play an improving role in the treatment of DR [31]. Hence, AVL had an inhibitory effect on AR, which can be considered as a potential efficacious alternative for intervening in DR.

### 3.2. The Effect of AVL on Cell Viability

The viability of ARPE-19 cells treated with AVL (6.25–100 µg/mL) in high-glucose condition (HG, 30 mM) was shown in Figure S1A. Notably, AVL did not exhibit significant cytotoxicity, but the cell viability slightly decreased with the increase in its concentration.

### 3.3. The Effect in the Polyol Pathway

The polyol pathway is a glucose metabolism pathway, which is considered to be an important factor in the pathogenesis of diabetic eye refractive changes, cataract formation, and diabetic retinopathy [32]. The concentration and action time of AVL for treating ARPE-19 cells induced by high-glucose condition were established according to the pre-experiment in the supplementary material (Figure S2A–D).

#### 3.3.1. The Effect of AVL on AR

**Figure 2:**
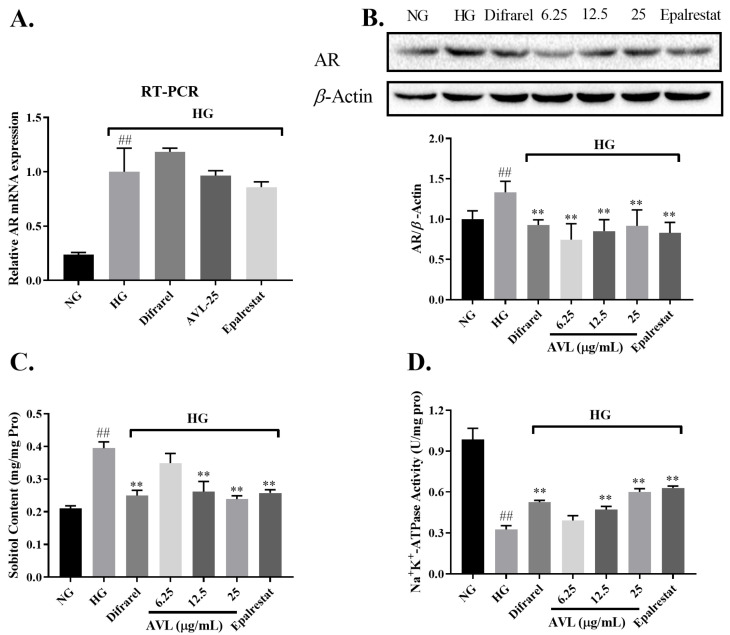
The effect of AVL on the polyol pathway in high glucose-induced ARPE-19 cells: (**A**) The relative expression of the AR gene by quantitative real-time PCR. (**B**) The expression of protein on AR by Western blotting. (**C**) The effect of AVL on sorbitol content. (**D**) The effect of AVL on Na^+^K^+^-ATPase activity. *n* = 3 independent experiments. A *p*-value < 0.05 was considered as a significant difference between each group (## *p* < 0.01 versus NG; ** *p* < 0.01 versus HG).

Aldose reductase, the first rate-limiting enzyme involved in the polyol pathway, catalyzes the conversion of glucose to sorbitol. The results showed that HG treatment significantly up-regulated AR mRNA (Figure 2A) and protein transcriptional levels (Figure 2B) in ARPE-19 cells, while 25 µg/mL AVL had no effect on AR mRNA level, but it down-regulated AR protein expression. This result was consistent with the positive drugs that AVL blocks AR expression at the protein level and not at the transcriptional level [33]. 

#### 3.3.2. The Effect of AVL on Sorbitol Content

Sorbitol is a molecule that poorly penetrates cell membranes, and its accumulation creates hyperosmotic stress. As shown in Figure 2C, the sorbitol content in HG group was significantly increased to 0.39 mg/mg protein (*p* < 0.01), it was reduced by treatment with AVL in a dose-dependent manner when the AVL concentration was 12.5 µg/mL (0.26 mg/mg protein, *p* < 0.01), the intracellular sorbitol level is very close to it in positive groups, and when curative concentration arrived to 25 µg/mL (0.24 mg/mg protein, *p* < 0.01), the content of sorbitol was lower than positive groups (Difrarel, Epalrestat; 0.25 mg/mg protein, 0.26 mg/mg protein; *p* < 0.01, *p* < 0.01). 

#### 3.3.3. The Effect of AVL on Na^+^ K^+^-ATPase Activity

Na^+^ K^+^-ATPase is an ion transporting enzyme that exchanges Na^+^ for K^+^ by hydrolyzing ATP. The effect of AVL and positive groups on Na^+^ K^+^-ATPase in HG-injured ARPE-19 cells is shown in Figure 2D. The activity of Na^+^ K^+^-ATPase was significantly decreased in ARPE-19 cells treated in the HG group (NG vs. HG, 0.49 vs. 0.16 U/mg protein), and AVL dose-dependently enhanced Na^+^ K^+^-ATPase activity. At a concentration of 12.5 µg/mL (0.24 U/mg protein, *p* < 0.01), the Na^+^ K^+^-ATPase activity was close to the effect of positive drug of Difrarel (0.26 U/mg protein, *p* < 0.01), and at a concentration of 25 *µ*g/mL (0.30 U/mg protein, *p* < 0.01), it was similar to the effect of epalrestat (0.31 U/mg protein, *p* < 0.01).

Under hyperglycemia, glucose flux, which enters the polyol pathway, is increased due to the saturation of hexokinase with glucose. Aberrant activation of aldose reductase drives the massive production of sorbitol, which is beyond the capacity of sorbitol dehydrogenase [9,32]. The accumulation of sorbitol creates osmotic swelling and cell dysfunction and decreases Na^+^ K^+^-ATPase. In previous studies, the structure–activity relationship of dietary polyphenol in inhibiting aldose reductase (AR) was reported [34]; Vitamin E could prevent the loss of Na^+^ K^+^-ATPase, thus significantly reducing the incidence of cataract [35]; Eparastat was also a common AR inhibitor and reduced aldose reductase in ARPE-19 cells under high-glucose condition [25]; the main bio-ingredients of Difrarel were anthocyanin extract of *Vaccinium myrtillus* L., belonging to the flavonoid class of compounds, which were used to prevent and improve visual fatigue and early stage of diabetes retinopathy[36]. We also found that AVL was useful for improving human retinal pigment epithelium cell damage induced by high glucose via decreasing the content of sorbitol, improving the activity of Na^+^ K^+^-ATPase, and down-regulating AR protein expression to regulate the polyol pathway. 

### 3.4. The Effect of AVL on Glutathione (GSH) and Reactive Oxygen Species (ROS)

Excessive levels of glucose stimulate oxidative stress by multiple mechanisms, and the polyol pathway is an important mechanism. Due to the overactivity of the polyol pathway, the excessive consumption of NADPH reduces the content of glutathione (GSH) during the conversion of glucose to sorbitol, and production of NADH increases reactive oxygen species (ROS) during the conversion of sorbitol to fructose [9].

**Figure 3:**
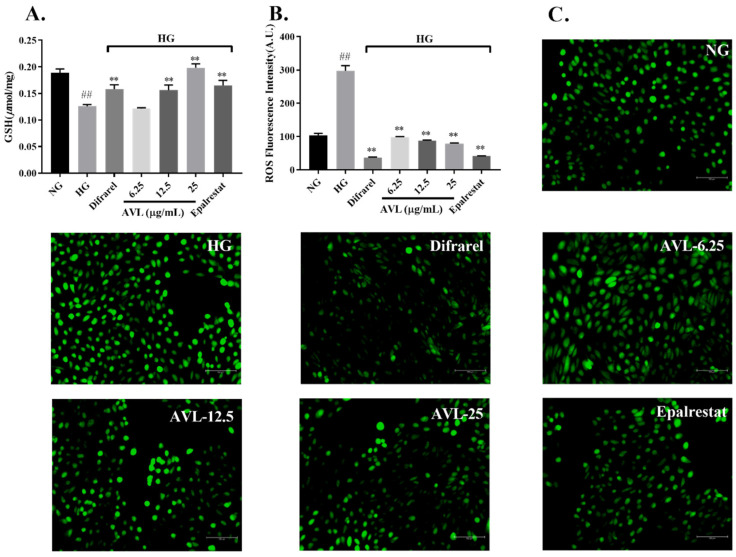
The effect of AVL on the GSH and ROS in high glucose-induced ARPE-19 cells: (**A**) The effect of AVL on intracellular glutathione (GSH) levels. (**B**) The effect of AVL on intracellular reactive oxygen species (ROS) content. (**C**) The effect of AVL on DCFH-DA staining for ROS. *n* = 3 independent experiments. A *p*-value < 0.05 was considered as a significant difference between each group (## *p* < 0.01 versus NG; ** *p* < 0.01 versus HG).

It has been observed (Figure 3A) that the glutathione decreased significantly after high-glucose injury (NG vs. HG, 0.19 vs. 0.13 µmol/mg Protein; *p* < 0.01), and increased in dose dependence manner after treated by AVL and at concentrations of 12.5 µg/mL and 25 µg/mL, GSH levels in ARPE-19 cells increased significantly (0.16 µmol/mg Protein, 0.20 µmol/mg Protein; *p* < 0.01, *p* < 0.01), similar result was also found in positive groups (Difrarel, Epalrestat; 0.16 µmol/mg Protein, 0.17 µmol/mg Protein; *p* < 0.01, *p* < 0.01). 

The intracellular ROS level was reduced remarkably by AVL intervention based on DCFH-DA staining of microplate spectrophotometer in Figure 3B (6.25 µg/mL, 12.5 µg/mL, 25 µg/mL; 98.05 A.U., 87.63 A.U., 79.12 A.U.; *p* < 0.01, *p* < 0.01, *p* < 0.01), similar with positive-treated groups (Difrarel, Epalrestat; 36.98 A.U., 41.04 A.U.; *p* < 0.01, *p* < 0.01). The enhancement of ROS level in ARPE-19 cells challenged by high glucose was validated [37] and was reduced by AVL based on DCFH-DA staining of fluorescence microscopy in Figure 3C. 

The GSH was decreased, and ROS were increased obviously in APRE-19 cells damaged with the high-glucose condition, which resulted in oxidative stress and accelerated DR process [38]. Dietary polyphenols are important natural antioxidants with a wide range of sources. The extract of green tea protected the retina against diabetic retinopathy via an antioxidant mechanism and was rich in polyphenol components [37]. The protective effects of AVL were also evaluated in ARPE-19 cells under the high-glucose condition, and these results suggested that AVL increased intracellular GSH levels and decreased ROS levels to protect the retina against oxidative stress caused by the polyol pathway.

### 3.5. AVL Reduces the Expression of Autophagic Proteins in High-Glucose-Treated ARPE-19 Cells

Autophagy is an important intracellular homeostasis process; abnormal activation of the polyol pathway, oxidative stress, cellular accumulation of ROS, and other stress responses play an important role in the triggering of autophagy [39].

The biogenesis of autophagosomes is orchestrated by autophagy-related (ATG) proteins, which first produce phagosomes in hierarchical order and then expand them into autophagosomes. In the initial stage of autophagy, induction of autophagy leads to the formation of autophagosomes mediated by the Vps-Beclin-1 complex, which initiates cell membrane vesicle nucleation. Autophagosome extension requires the involvement of ATG12 and LC3. ATG12 is activated by ATG7 and transported to ATG5 to form the ATG5-ATG12 complex, which further forms ATG5-ATG12-ATG16 to participate in the expansion of autophagosome phagocytic vesicles. In addition, LC3B-I is activated and transferred to ATG3 to form LC3-II-PE with phosphatidylethanolamine (PE) to participate in membrane extension; Phagocytosis and lysosomes fuse to form mature autophagosomes and degrade their contents [40,41]. Hence, the expression of key autophagic proteins in high-glucose-treated ARPE-19 cells is analyzed to evaluate the effect of AVL on autophagy levels.

**Figure 4:**
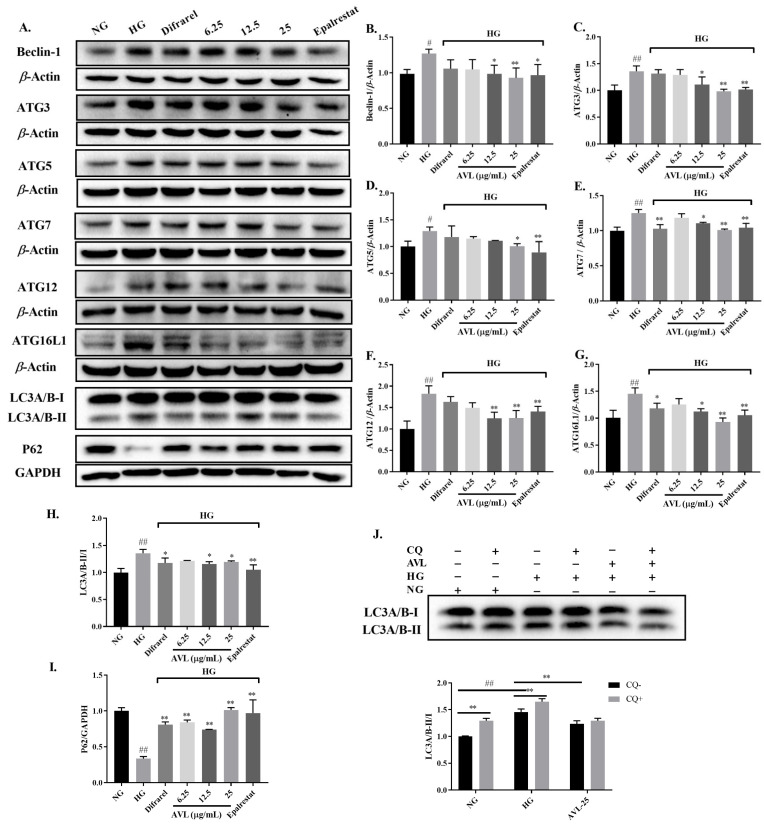
AVL regulate autophagic protein expression in ARPE-19 cells under high-glucose stress. (**A**) Representative protein gel blots of Beclin-1, ATG3, ATG5, ATG7, ATG12, ATG16L1, LC3 and P62 are shown. The *β*-Actin and GAPDH were utilized as the loading control. (**B**–**I**) The histogram of protein content of Beclin-1, ATG3, ATG5, ATG12, ATG16L1, LC3 and p62. (**J**) Densitometric analysis of LC3 in ARPE-19 treated with CQ. *n* = 3 independent experiments. A *p*-value < 0.05 was considered as a significant difference between each group (# *p* < 0.05, ## *p* < 0.01 versus NG; * *p* < 0.05, ** *p* < 0.01 versus HG).

To expound the mechanism of AVL inhibits increased autophagy in the HG group, the expression of significant autophagy proteins Beclin-1, ATG3, ATG5, ATG7, ATG12, ATG16L1, LC3, and P62 were examined by Western Blot in Figure 4A–I. The expression of autophagy-related proteins was significantly increased in ARPE-19 cells treated with high glucose (HG group), indicating that high glucose treatment increased the level of autophagy. However, the protein expressions of Beclin-1 (12.5 µg/mL, 25 µg/mL; *p* < 0.05, *p* < 0.01), ATG3 (12.5 µg/mL and 25 µg/mL; *p* < 0.05, *p* < 0.01), ATG5 (25 µg/mL; *p* < 0.05), ATG7 (12.5 µg/mL and 25 µg/mL; *p* < 0.05, *p* < 0.01), ATG12 (12.5 µg/mL and 25 µg/mL; *p* < 0.01, *p* < 0.01), ATG16L1 (12.5 µg/mL and 25 µg/mL; *p* < 0.05, *p* < 0.01), and LC3-II/LC3-I (12.5 µg/mL and 25 µg/mL; *p* < 0.05, *p* < 0.05) were diminished and P62 (6.25 µg/mL, 12.5 µg/mL and 25 µg/mL; *p* < 0.01, *p* < 0.01, *p* < 0.01) was increased significantly after treated by AVL. The mechanism of AVL was consistent with that of epalrestat, but the Difrarel group only significantly improved the protein expression of Atg7, ATG16L1, LC3-II/LC3-I, and P62. This indicated that autophagy stages of elongation and maturation were influenced by Difrarel, which was not fully consistent with AVL. The accumulation of autophagy proteins was due to the increase of autophagy initiation or the disorder of fusion between autophagosome and lysosome under high glucose conditions. The level of the P62 protein, negatively correlated with autophagy activity, was combined with the ratio of LC3-II/LC3-I to evaluate the level of autophagy. Autophagy plays a protective and repair role under normal conditions, while dysregulation of autophagy could mediate the damage of high glucose to ARPE-19 cells directly [39]. This result is consistent with Gao et al.; they found that the level of intracellular autophagy was increased after high glucose injury in ARPE-19 cells, and *Lycium barbarum polysaccharide* can decrease the autophagy levels of ARPE-19 cells [39,42]. In addition, it has been reported that *A. venetum* leaf extract has a positive effect on injured neurons by regulating the levels of autophagy and apoptosis in PC12 cells [16]. Our result demonstrates that AVL reduced autophagy caused by the high-glucose condition via down-regulation of the over-activated autophagic proteins to maintain autophagy homeostasis in ARPE-19 cells.

#### 3.5.1. Measurement of Autophagic Flux

LC3-I is converted into LC3-II, which directly participates in autophagosome formation and elongation and is considered an important autophagy marker. Autophagy is a highly dynamic pathway; chloroquine (CQ) is an autophagy inhibitor that blocks the last stage of autophago–lysosomal fusion and inhibits the degradation of LC3-II from lysosomes in the cytoplasm [43]. When autophagy is activated, a CQ is added, LC3-II degradation is inhibited, and the expression of LC3-II is higher than without the inhibitor. Hence, the autophagic fluxn is evaluated by LC3-II/LC3-I ratio in the presence or absence of the autophagy inhibitor CQ.

The previous study has shown that inhibition of the autophago–lysosomal fusion by CQ significantly increased the LC3-II/LC3-I ratio and, thus, autophagic flux in H_2_O_2_-treated ARPE-19 and hRPE cells [41]. ARPE-19 Cells in the CQ + group were treated with 10 µM CQ for 1 h before glucose treatment. There was a significant difference between the HG group and HG+CQ group on the level of LC3-II/LC3-I (*p* < 0.01) in Figure 4J. When autophagy activity was enhanced, the expression of LC3-II/LC3-I was higher than before the addition of the CQ, indicating that high-glucose stimulated the activation of autophagic flux. Additionally, there was no significant difference between the LC3-II/LC3-I in cells treated with AVL alone or combined with CQ (AVL group vs. AVL + CQ group), indicating that AVL can inhibit the initial stage of autophagy to protect ARPE-19cells from high glucose injury.

#### 3.5.2. AVL Inhibit Upstream Autophagy Induced by High Glucose in ARPE-19 Cells via the AMPK/mTOR/ULK1 Signaling Pathway

The AMPK/mTOR/ULK1 signaling pathway is the switch of intracellular anabolic and catabolic processes and is also an important regulatory pathway upstream of autophagy.

**Figure 5:**
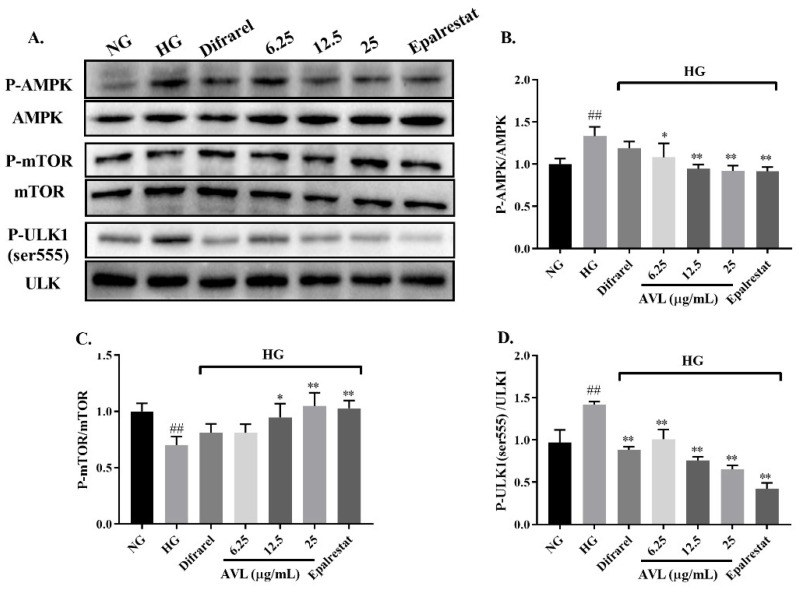
The effect of AVL on the AMPK/mTOR/ULK1 pathway in ARPE-19 cells under high-glucose condition: (**A**) Representative protein gel blots of P-AMPK/AMPK, P-mTOR/mTOR and P-ULK (ser555)/ULK. (**B**–**D**) The histogram of protein content of P-AMPK/AMPK, P-mTOR/mTOR and P-ULK (ser555)/ULK. *n* = 3 independent experiments. A *p*-value < 0.05 was considered as a significant difference between each group (## *p* < 0.01 versus NG; * *p* < 0.05, ** *p* < 0.01 versus HG).

AMP-activated protein kinase (AMPK) is an energy-sensitive kinase that monitors the ratio of AMP/ATP; the mammalian target protein of rapamycin (mTOR) regulates cellular processes and unc-51-like kinase 1 (ULK1) is the initial switch of autophagy. The level of AMPK, mTOR, and ULK1 proteins in ARPE-19 cells is clarified in Figure 5A–C. The expression level of P-AMPK(Thr172)/AMPK and *p*-ULK1(ser555)/ULK increased, and the level of P-mTOR/mTOR decreased in ARPE-19 cells with the high-glucose condition (HG group) compared with the NG group, markedly. However, AVL significantly reduced the expressions of P-AMPK/AMPK (6.25 µg/mL, 12.5 µg/mL and 25 µg/mL; *p* < 0.05, *p* < 0.01, *p* < 0.01) and *p*-ULK1/ULK1 (6.25 µg/mL, 12.5 µg/mL and 25 µg/mL; *p* < 0.01, *p* < 0.01, *p* < 0.01) compared with the HG group. The level of P-mTOR/mTOR was greatly increased in the AVL group (12.5 µg/mL, 25 µg/mL; *p* < 0.05, *p* < 0.01) compared with the HG group, which is consistent with the positive group of epalrestat. However, this finding was different from the result of the Difrarel group in that only the protein level of P-ULK1/ULK1 was inhibited compared with the HG group, indicating that Difrarel may act through additional signaling pathways to improve DR, such as the inflammatory/antioxidative signaling [44]. In addition, it is also possible that the dosage of Difrarel is insufficient. Therefore, the mechanism of Difrarel in the treatment of DR can be further explored.

The AMPK activated by phosphorylation of Thr172 promoted phosphorylation of the rapamycin target site on mTORC1 to inhibit the activity of mTORC1, thereby activating the Ser555 site of ULK1 and finally inducing autophagy; in addition, AMPK can also directly phosphorylate ULK1 to initiate autophagy [45]. Autophagy was up-regulated in vascular smooth muscle cells (VSMCs) of DM via activation of the AMPK/mTOR pathway, and epigallocatechin-3-gallate from green tea can inhibit autophagy against cardiomyopathy in diabetic Goto-Kakizaki rats [46,47]. And when ARPE-19 cells were treated with a high glucose condition (30 mM) for 48 h, the p-AMPK/AMPK ratio was higher than the normal glucose condition, LC3 II expression was significantly increased, and autophagy was activated [22]. Additionally, the phosphorylation level of mTOR was down-regulated in autophagy-activated ARPE-19 cells under high glucose conditions [39]. Thus, AVL decreased the phosphorylation of AMPK, increased mTOR activity, and decreased ULK1 activity, regulating the level of autophagy finally.

### 3.6. The Effect of AVL on Cellular Morphology

**Figure 6:**
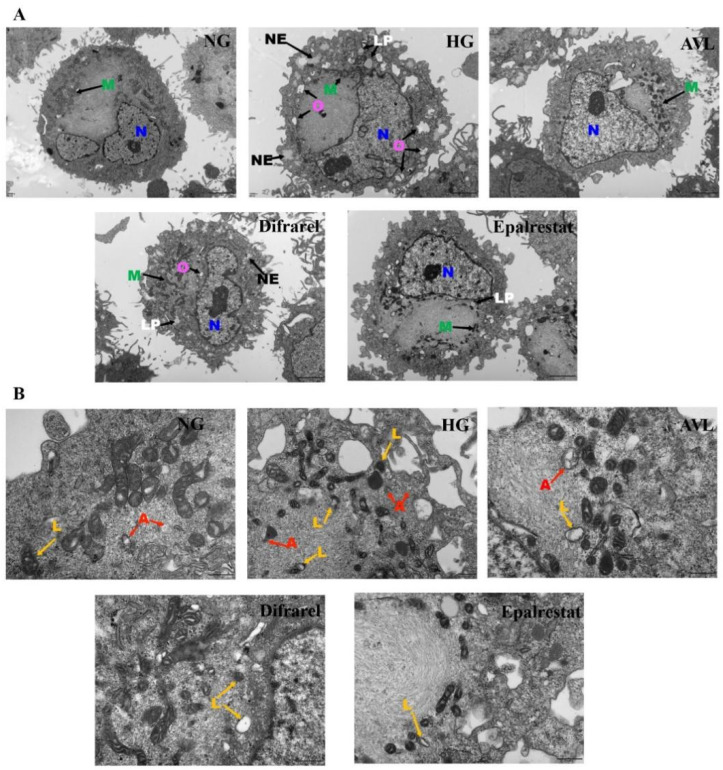
Observation of ARPE-19 cells under transmission electron microscope. (**A**) Transmission electron microscope to observe the intracellular morphology. N: nucleus; M: mitochondria; O: oedema; NE: necrosis; LP: lipid droplets. (**B**) The autophagic vesicles on transmission electron microscope. A: autophagosomes, L: autolysosome.

The cellular morphology of ARPE-19 cells was observed by transmission electron microscopy (TEM). The ultrastructural features are shown in Figure 6A; lipid droplets, necrosis, edema, and a blurred mitochondrial outline were observed when cells were exposed to high glucose treatment for 48 h, which suggests that cell damage was clear in the HG group [40,48]. After AVL (25 µg/mL) and positive group treatment, these damage conditions were relieved with significantly reduced lipid droplets, necrosis, and edema. This observation indicated that AVL and positive groups could attenuate the damage of retina cells caused by high glucose and have a protective effect. Furtherly, the autophagy state of ARPE-19 cells was observed in Figure 6B. Autophagosomes are mainly composed of double membranes, and a few of them are closed circular structures formed by multilayer or monolayer membranes, which are surrounded by damaged organelles or proteins; autophagic lysosomes are formed by the fusion of autophagosome and lysosome, with monolayer membrane structure, which is surrounded by degraded cytoplasmic components [22]. Autophagosomes and autophagic lysosomes were observed in the HG group but decreased in the AVL group and positive treatment group. These results suggested that AVL could protect the cellular morphology and restore the autophagy level of high glucose-injured ARPE-19 cells.

ARPE-19 cells with long-term exposure to high glucose conditions trigger abnormal polyol pathways with the accumulation of sorbitol and decreased Na^+^ K^+^-ATPase, oxidative stress with the decrease of GSH, and the generation of ROS, resulting in activating autophagy eventually. AVL can restore the polyol pathway via inhibiting AR, protecting ARPE-19 cells from oxidative stress. The AMPK/mTOR/ULK1 signaling pathway, the switch of anabolic and catabolic processes, is an important regulatory pathway upstream of autophagy. Previously reported that AR regulated ROS/SIRT1/AMPK/mTOR pathway to protect human umbilical vascular endothelial cells (HUVECs) from dysfunction [49], Fidarestat, as an AR inhibitor, regulated the expression of AMPK, mTOR, and P53 to prevent colorectal cancer cell (CRC) growth [50]. The AR correlated with AMPK/mTOR pathway and disrupted autophagy, and AR inhibitor epalrestat is neuroprotective against brachial plexus root avulsion injury through the regulation of autophagy to alleviate neuroinflammation and rescue massive motoneurons death in mice [51]. Therefore, AVL could regulate AR and the AMPK/mTOR/ULK1 pathway to maintain intracellular autophagy and protect ARPE-19 cells from high glucose conditions.

## 4. Conclusions

AVL enriched polyphenolic compounds, including sixty-eight flavonoids, twenty-one organic acids, and four coumarins, which can effectively inhibit aldose reductase in vitro. AVL could decrease the protein level of AR and sorbitol content and enhance the Na^+^ K^+^-ATPase activity to improve the over-activated polyol pathway caused by high glucose condition; it also could be used against retina oxidative stress through increasing intracellular GSH level and reducing ROS level. Furthermore, AVL could inhibit hyper-autophagy in ARPE-19 cells, which is induced by high glucose conditions through the AMPK/mTOR/ULK1 signaling pathway, to alleviate the APRE-19 cell damage caused by high glucose. Collectively, AVL could be an in vitro aldose reductase inhibitor that restores the polyol pathway, inhibits oxidative stress, and maintains intracellular autophagy through the AMPK/mTOR/ULK1 pathway, which can be a potential mechanism for the AVL treatment of DR. Hence, the polyphenol-rich extract of *A. venetum* leaves is a promising functional food and candidate pharmaceutical for the prevention and the early treatment of DR. This study revealed the pharmacodynamic material basis and the protective mechanism of *A. venetum* leaves against DR, which provided a scientific basis for further research. However, there was a limitation in our study. We only conducted experiments in cells but did not verify our results in animals. We will further verify our results in animals in future experiments.

## Figures and Tables

**Table 1:** Identification of AVL using UHPLC-Q-Orbitrap-HRMS.

No.	t_R_ (min)	Molecular Formula	Molecular Ion (*m*/*z*)	Ion Mode	Error (ppm)	MS^2^ Data (*m*/*z*)	Identification
1	1.76	C_15_H_14_O_6_	288.9369	[M−H]-	-	261(5), 245(5), 175(100), 159(50), 147(80), 131(60)	Catechin/Epicatechin
2	1.82	C_15_H_14_O_7_	305.0037	[M−H]-	-	175(100), 159(10), 147(90), 131(10)	Gallocatechin/Epigallocatechin
3	10.9	C_30_H_26_O_13_	593.1296	[M−H]-	1.1321	467(20), 423(90), 305(20), 289(60), 125(100)	Gallocatechin-(4,8)-catechin
4	13.66	C_30_H_26_O_13_	593.131	[M−H]-	3.4989	467(10), 425(40), 407(70), 303(10), 289(60), 245(20), 177(100), 167(10), 125(90)	Gallocatechin-(4,8)-catechin
5	16.15	C_30_H_26_O_12_	577.1385	[M−H]-	−2.3839	451(20), 425(40), 407(70), 289(80), 245(20), 125(100)	Procyanidin B1
6	16.53	C_15_H_14_O_7_	305.0668	[M−H]-	4.2491	261(10), 243(5), 203(5), 167(40), 137(40), 125(100)	Gallocatechin
7	17.45	C_30_H_26_O_13_	593.1315	[M−H]-	4.3221	467(20), 425(60), 407(20), 305(60), 289(30), 245(20), 177(50), 125(100)	Catechin-(4,8)-Gallocatechin
8	19.7	C_27_H_30_O_17_	625.1422	[M−H]-	3.7547	463(50), 299(100), 271(70), 243(5), 151(5)	Baimaside
9	20.96	C_27_H_30_O_17_	625.1422	[M−H]-	3.7547	463(50), 299(100), 271(70), 243(5), 151(5)	Quercetin-3-O-sophoroside
10	21.24	C_33_H_40_O_21_	771.2004	[M−H]-	−4.2288	609(50), 462(30), 299(100), 271(70), 243(5), 151(5)	Rutin-glucoside
11	22.3	C_18_H_26_O_10_	401.146	[M−H]-	4.5062	269(100), 161(60)	Apigenin-arabinoside
12	23.57	C_30_H_26_O_12_	577.1361	[M−H]-	3.5621	451(20), 425(40), 407(90), 289(80), 245(20), 125(100)	Procyanidin B2
13	25.22	C_27_H_30_O_16_	609.146	[M−H]-	2.1185	447(80), 327(5), 285(100), 255(60), 151(10)	Kaempferol-3-O-sophoroside
14	25.29	C_29_H_32_O_18_	667.1525	[M−H]-	1.5909	505(30), 463(30), 299(100), 271(70), 243(5), 151(5)	Acetylhyperoside
15	25.96	C_15_H_13_O_6_	289.0719	[M−H]-	-	245(60), 203(40), 179(20), 123(50)	Catechin/Epicatechin
16	26.54	C_29_H_32_O_18_	667.1525	[M−H]-	3.144	505(20), 463(30), 299(100), 271(60), 243(10), 151(5)	AcetylIsoquercitrin
17	28.51	C_21_H_18_O_13_	477.0687	[M−H]-	4.91441	301(100), 287(10), 271(5), 179(20), 151(40)	Quercetin-O-glucuronide
18	29.33	C_21_H_20_O_13_	479.0841	[M−H]-	4.4887	316(90),299(100),287(20),271(90),179(20)	Myricetin-3-O-galactoside
19	31.81	C_27_H_30_O_17_	625.1423	[M−H]-	3.8524	463(90), 317(10), 300(100), 271(60), 255(40), 243(20), 179(20), 151(40)	Quercetin-O-diglucoside
20	33.55	C_21_H_20_O_13_	479.0839	[M−H]-	4.0429	316(100), 301(10), 287(30), 271(50), 179(20), 151(10)	Myricetin 3-O-glucoside
21	34.15	C_21_H_20_O_13_	479.0841	[M−H]-	4.3614	316(100), 287(40), 271(50), 179(10), 151(5)	Myricetin 3-O-glucoside-isomer
22	35.44	C_21_H_18_O_13_	477.0635	[M−H]-	4.565	301(100), 273(10), 255(5), 179(20), 151(40)	Quercetin-O-glucuronide
23	36.25	C_21_H_18_O_13_	477.0655	[M−H]-	4.6602	301(100), 273(10), 255(5), 179(20), 151(40)	Quercetin-O-glucuronide
24	37.51	C_27_H_30_O_16_	609.147	[M−H]-	3.4206	300(100), 271(60), 255(30), 227(10), 179(10), 151(10)	Rutin-isomer
25	37.71	C_15_H_12_O_6_	289.0708	[M+H]-	0.4711	179(10), 171(40), 163(50), 153(100), 145(5), 135(20)	Eriodictyol
26	37.99	C_27_H_30_O_16_	609.1471	[M−H]-	3.5208	300(100), 271(60), 255(30), 243(10), 227(10), 179(5), 151(15)	Rutin
27	38.71	C_21_H_20_O_12_	463.0893	[M−H]-	4.9312	301(100), 271(10), 227(10), 151(50)	Quercetin-O-glucoside
28	39.31	C_21_H_20_O_12_	463.0891	[M−H]-	4.4752	300(100), 271(70), 255(40), 227(10), 179(10), 151(15)	Hyperoside
29	39.31	C_23_H_22_O_13_	505.0996	[M−H]-	3.8454	463(5), 300(100), 271(80), 255(40), 243(20), 179(10), 151(15)	Quercetin-3-O-[6′′-O-acetyl]-galactoside
30	39.65	C_24_H_22_O_15_	549.0981	[M−H]-	4.6315	505(80), 300(100), 271(70), 255(40), 151(20)	Quercetin-3-O-[6′′-O-malonyl]-galactoside
31	39.95	C_21_H_20_O_12_	463.0889	[M−H]-	3.8899	300(100), 271(80), 255(40), 243(20), 179(5), 151(20)	Isoquercitrin
32	40.71	C_27_H_30_O_15_	595.1654	[M+H]-	−0.4708	449(5), 287(100)	Cyanidin-3-rutinoside
33	40.87	C_24_H_22_O_15_	549.0893	[M−H]-	3.9338	505(90), 300(100), 271(60), 255(40), 229(15), 151(20)	Quercetin-3-O-[6′′-O-malonyl]-glucoside
34	40.9	C_27_H_30_O_15_	593.1516	[M−H]-	2.6568	284(80), 255(60), 227(30), 151(5)	Kaempferol-3-O-rutinoside
35	41.63	C_21_H_18_O_13_	477.0683	[M−H]-	4.1032	301(100), 255(10), 227(50), 179(20), 151(40)	Quercetin-O-glucuronide
36	41.91	C_29_H_32_O_17_	651.158	[M−H]-	3.7537	609(40), 463(10), 301(100), 271(70), 255(40), 227(10), 179(10), 151(20)	Acetyled rutin
37	42.19	C_20_H_18_O_11_	433.0776	[M−H]-	4.0175	300(100), 271(80), 255(50), 243(20), 179(10), 151(5)	Quercetin-O-arabinoside
38	42.26	C_29_H_32_O_17_	593.152	[M−H]-	−2.6433	285(90), 255(50), 227(30), 151(5)	Kaempferol-3-O-rutinoside
39	42.46	C_29_H_32_O_17_	593.152	[M−H]-	3.2742	285(90), 255(50), 227(30)	Kaempferol-7-O-rutinoside?
40	42.51	C_27_H_30_O_15_	595.1646	[M+H]-	−1.804	287(100)	Cyanidin-3-rutinoside
41	42.73	C_24_H_22_O_15_	549.09	[M−H]-	4.6315	505(90), 300(100), 271(60), 255(40), 229(15), 151(20)	Quercetin-3-O-[6′′-O-malonyl]-glucoside
42	42.81	C_46_H_24_O_3_	623.1627	[M−H]-	−2.329	463(20), 314(40), 299(50), 271(40), 243(20), 151(10)	Narcissoside
43	42.84	C_28_H_24_O_16_	593.1522	[M−H]-	3.3035	285(100), 255(50), 227(30)	Nicotiflorin
44	42.98	C_21_H_20_O_11_	447.0943	[M−H]-	4.791	284(70), 255(80), 227(60)	Cynaroside
45	43.44	C_28_H_24_O_16_	623.1625	[M−H]-	3.0096	315(100), 300(50), 255(20), 243(30), 151(10)	Narcissoside-isomer
46	44.04	C_23_H_22_O_13_	505.0996	[M−H]-	4.0097	463(5), 300(100), 271(70), 255(30), 227(5), 179(10), 151(20)	Quercetin-3-O-[6′′-O-acetyl]-glucoside-isomer
47	44.14	C_24_H_22_O_15_	549.089	[M−H]-	2.4849	505(95), 300(100), 271(80), 255(50), 243(20), 151(10)	Quercetin-3-O-[6′′-O-malonyl]-glucoside
48	44.18	C_21_H_20_O_12_	463.0892	[M−H]-	4.7335	300(100), 271(70), 255(30), 227(5), 179(10), 151(20)	Quercetin-O-glucoside
49	44.65	C_21_H_20_O_11_	447.0939	[M−H]-	3.8354	284(70), 255(90), 227(80), 151(5)	Kaempferol-O-glucoside
50	44.69	C_15_H_12_O_5_	271.0616	[M−H]-	-	151(80), 119(90)	Naringenin/Butein/7,3′,4′-trihydroxyflavanone
51	44.9	C_23_H_22_O_12_	489.104	[M−H]-	2.5705	447(5), 284(100), 255(70), 227(20), 191(5)	Kaempferol-3-O-[6′′-O-acetyl]-galactoside
52	46.04	C_21_H_20_O_12_	463.0881	[M−H]-	2.2293	301(100), 285(50), 255(10), 229(5), 179(30), 151(50)	Quercetin-O-glucoside
53	46.52	C_21_H_18_O_13_	477.1046	[M−H]-	4.9897	449(5), 314(20), 299(100), 271(60), 243(5), 199(5), 151(10)	Quercetin-O-glucuronide
54	46.72	C_24_H_22_O_15_	549.0895	[M−H]-	3.7117	505(80), 300(100), 271(70), 255(40), 243(10), 151(10)	Quercetin-3-O-[6′′-O-malonyl]-glucoside
55	47.18	C_23_H_22_O_12_	489.104	[M−H]-	2.6695	285(100), 255(80), 227(40), 151(5)	Kaempferol-3-O-[6′′-O-acetyl]-galactoside
56	47.39	C_24_H_22_O_14_	533.0944	[M−H]-	3.5667	489(50), 285(100), 255(50), 227(30)	Kaempferol-3-O-[6′′-O-malonyl]-galactoside
57	47.39	C_23_H_22_O_12_	489.1039	[M−H]-	2.4109	285(100), 255(80), 227(40), 151(5)	Kaempferol-3-O-[6′′-O-acetyl]-galactoside
58	47.99	C_15_H_10_O_8_	317.0304	[M−H]-	3.9834	299(5), 255(5), 179(40), 151(80)	Myricetin
59	48.17	C_23_H_22_O_12_	489.1041	[M−H]-	2.9108	284(100), 255(80), 227(40), 151(5)	Kaempferol-3-O-[6′′-O-acetyl]-galactoside
60	48.49	C_21_H_24_O_10_	435.1307	[M−H]-	4.9031	273(100), 179(10), 167(90), 123(40)	Dihydromyricetin-O-glucoside
61	48.86	C_24_H_22_O_15_	549.0894	[M−H]-	3.4276	505(80), 300(100), 271(50), 255(30), 179(20), 151(10)	Quercetin-3-O-[6′′-O-malonyl]-glucoside
62	49.23	C_15_H_10_O_8_	317.0301	[M−H]-	3.1171	271(5), 179(40), 151(60)	Myricetin
63	49.34	C_21_H_20_O_8_	463.0894	[M−H]-	5.063	301(100), 179(30), 151(70)	Quercetin-O-glucoside
64	49.93	C_24_H_22_O_14_	533.0943	[M−H]-	3.2212	489(70), 463(20), 285(100), 255(70), 227(40)	Kaempferol-3-O-[6′′-O-malonyl]-glucoside
65	50.04	C_23_H_22_O_12_	489.1041	[M−H]-	2.7578	285(100), 255(80), 227(50)	Kaempferol-3-O-[6′′-O-acetyl]-galactoside
66	52.63	C_15_H_10_O6	287.0554	[M+H]-	−1.8834	258(5), 227(5), 153(20), 121(10)	Cyanidin
67	61.28	C_15_H_9_O_7_	301.0348	[M−H]-	1.9001	273(10), 229(5), 179(40), 151(90)	Quercetin
68	67.92	C_15_H_12_O_5_	271.0615	[M−H]-	5.2516	227(5), 151(90), 119(100)	Naringenin
69	2.6	C_15_H_18_O_9_	341.1077	[M−H]-	−0.2353	179(50), 119(60), 89(100)	Caffeoylglucopyranose
70	2.94	C_7_H_11_O_6_	191.0561	[M−H]-	-	176(20), 162(50), 144(100)	Quinic acid
71	6.67	C_7_H_6_O_5_	169.0143	[M−H]-	-	125(100)	Gallic acid
72	12.02	C_28_H_38_O_19_	677.1934	[M−H]-	1.5731	353(10), 191(100), 179(10), 135(10)	Dicaffeoylquinic acid glucoside
73	12.05	C_21_H_28_O_14_	503.142	[M−H]-	4.4169	341(70), 179(40), 161(100), 135(30)	Caffeoyl diglucoside
74	13.33	C_16_H_18_O_9_	353.0867	[M−H]-	−1.021	191(100), 179(70), 135(60)	3-O-caffeoylquinic acid
75	16.06	C_17_H_20_O_9_	367.1032	[M−H]-	2.4036	191(100), 173(10), 135(20)	3-O-feruloylquinic acid
76	16.89	C_17_H_20_O_9_	367.1041	[M−H]-	4.7313	191(100), 173(10), 135(20)	5-O-feruloylquinic acid
77	17.53	C_17_H_20_O_9_	367.104	[M−H]-	4.651	191(100), 173(10), 135(20)	4-O-feruloylquinic acid
78	20.12	C_16_H_18_O_9_	353.0857	[M−H]-	−2.7886	191(100)	5-O-caffeoylquinic acid
79	21.16	C_16_H_18_O_9_	353.0867	[M−H]-	−1.021	191(60), 179(70), 173(100), 135(80)	4-O-caffeoylquinic acid
80	27.82	C_16_H_18_O_8_	337.092	[M−H]-	0.666	191(100), 173(50), 163(10)	5-p-coumaroylquinic acid
81	31.99	C_16_H_18_O_8_	337.0931	[M−H]-	4.6521	191(100), 173(5)	4-p-coumaroylquinic acid
82	41.91	C_25_H_24_O_12_	515.0812	[M−H]-	3.9212	353(80), 191(100), 179(50), 173(6), 135(60)	1, 3-O-dicaffeoylquinic acid
83	42.33	C_25_H_24_O_12_	515.0812	[M−H]-	4.1581	353(80), 191(100), 179(70), 173(20), 135(60)	3, 4-O-dicaffeoylquinic acid
84	45.18	C_25_H_24_O_12_	515.0812	[M−H]-	3.6842	353(60), 191(100), 179(50), 173(20), 135(50)	3, 5-O-dicaffeoylquinic acid
85	45.71	C_25_H_24_O_12_	515.1205	[M−H]-	3.2102	353(90), 191(50), 179(70), 173(100), 135(70)	4, 5-O-dicaffeoylquinic acid
86	48.27	C_25_H_24_O_12_	515.1195	[M−H]-	2.9733	353(90), 191(40), 179(70), 173(100), 135(70)	1,5-O-dicaffeoylquinic acid
87	51.36	C_25_H_24_O_11_	499.183	[M−H]-	4.0078	337(50), 173(20), 163(100), 119(50)	p-coumaoylquinic acid glucoside
88	55.94	C_26_H_26_O_12_	529.1361	[M−H]-	4.0002	367(50), 193(20), 173(100), 135(70)	3-O-caffeoyl-4-O-feruloylquinic acid
89	56.58	C_26_H_26_O_12_	529.136	[M−H]-	3.8107	367(30), 353(70), 191(80), 179(40), 173(100), 135(70)	4-O-feruloyl-5-O-caffeoylquinic acid
90	14.92	C_15_H_16_O_9_	339.0721	[M−H]-	3.2902	177(100), 161(100), 149(5), 133(10), 105(5)	Esculin
91	18.47	C_9_H_8_O_7_	147.0443	[M+H]-	3.2643	119(950), 91(100), 65(20)	Coumarin
92	22.37	C_9_H_6_O_4_	177.0193	[M−H]-	6.4598	149(5), 133(40), 105(20)	Esculetin
93	27.85	C_18_H_12_O_7_	339.0498	[M+H]-	−0.305	147(100), 119(30), 91(20), 69(10)	Coumarin-glucoside

## Data Availability

The data presented in this study are available on request from the corresponding author. The data are not publicly available due to privacy.

## Supplementary Materials

The following supporting information can be downloaded at https://www.mdpi.com/article/10.3390/nu16172944/s1, Figure S1: Cytotoxicity of AVL to ARPE-19 cells for 24 h in high glucose (30 mM) (A). Verification of high glucose damage model on the relative expression of the AR gene by quantitative real-time PCR (B), sorbitol content (C), and Na^+^ K^+^-ATPase activity (D); Figure S2: Determination of AVL concentration. The high glucose model was treated with different concentration of AVL (6.25, 12.5, 25 and 50 μg/mL) on sorbitol content (A) and Na^+^ K^+^-ATPase activity (B). The high glucose model was treated with different time on AVL concentration of 25 μg/mL on sorbitol content (C) and Na^+^ K^+^-ATPase activity (D).

## References

[1] Cladis D.P., Weaver C.M., Ferruzzi M.G. (2022). (Poly)phenol toxicity in vivo following oral administration: A targeted narrative review of (poly)phenols from green tea, grape, and anthocyanin-rich extracts. Phytother. Res..

[2] Vitale M., Masulli M., Rivellese A.A., Bonora E., Cappellini F., Nicolucci A., Squatrito S., Antenucci D., Barrea A., Bianchi C. (2018). Dietary intake and major food sources of polyphenols in people with type 2 diabetes: The TOSCA.IT Study. Eur. J. Nutr..

[3] Quesada-Granados J.J., Rufián-Henares J.A., Chakradhari S., Sahu P.K., Sahu Y.K., Patel K.S. (2023). Comparative analysis of traditional oriental herbal fruits as potential sources of polyphenols and minerals for nutritional supplements. Molecules.

[4] Ruamviboonsuk P., Tiwari R., Sayres R., Nganthavee V., Hemarat K., Kongprayoon A., Raman R., Levinstein B., Liu Y., Schaekermann M. (2022). Real-time diabetic retinopathy screening by deep learning in a multisite national screening programme: A prospective interventional cohort study. Lancet Digit. Health.

[5] Shukla U.V., Tripathy K. (2023). Diabetic Retinopathy. StatPearls.

[6] Garcia-Aguilar A., Palomino O., Benito M., Guillen C. (2021). Dietary polyphenols in metabolic and neurodegenerative diseases: Molecular targets in autophagy and biological effects. Antioxidants.

[7] Akpoveso O.O.P., Ubah E.E., Obasanmi G. (2023). Antioxidant phytochemicals as potential therapy for diabetic complications. Antioxidants.

[8] Tang L., Xu G.T., Zhang J.F. (2023). Inflammation in diabetic retinopathy: Possible roles in pathogenesis and potential implications for therapy. Neural Regen. Res..

[9] Tang W.H., Martin K.A., Hwa J. (2012). Aldose reductase, oxidative stress, and diabetic mellitus. Front. Pharmacol..

[10] Dong L.Z., Li Y., Chen Q., Liu Y.H., Wu Z.F., Pan D.D., Yan N., Liu L.L. (2023). Cereal polyphenols inhibition mechanisms on advanced glycation end products and regulation on type 2 diabetes. Crit. Rev. Food Sci. Nutr..

[11] Peng J., Abdulla R., Li Y., Liu X.Y., He F., Xin X.L., Aisa H.A. (2023). Potential anti-diabetic components of *Apocynum venetum* L. flowers: Optimization, chemical characterization and quality evaluation. J. Food Compos. Anal..

[12] Shen J., Yang K., Jiang C., Ma X.Q., Zheng M.X., Sun C.H. (2020). Development and application of a rapid HPLC method for simultaneous determination of hyperoside, isoquercitrin and eleutheroside E in *Apocynum venetum* L. and *Eleutherococcus senticosus*. BMC Chem..

[13] Li C., Huang G., Tan F., Zhou X., Mu J., Zhao X. (2019). In Vitro analysis of antioxidant, anticancer, and bioactive components of *Apocynum venetum* Tea extracts. J. Food Qual..

[14] Zhang W., Dong Z., Chang X., Zhang C., Rong G., Gao X., Zeng Z., Wang C., Chen Y., Rong Y. (2018). Protective effect of the total flavonoids from *Apocynum venetum* L. on carbon tetrachloride-induced hepatotoxicity in vitro and in vivo. J. Physiol. Biochem..

[15] Irie K., Sato T., Tanaka I., Nakajima J., Kawaguchi M., Himi T. (2009). Cardiotonic effect of *Apocynum venetum* L. extracts on isolated guinea pig atrium. J. Nat. Med..

[16] Feng Y., Jiang C., Yang F., Chen Z., Li Z. (2020). *Apocynum venetum* leaf extract protects against H2O2-induced oxidative stress by increasing autophagy in PC12 cells. Biomed. Rep..

[17] Yuan Y., Zhou J., Zheng Y., Xu Z., Li Y., Zhou S., Zhang C. (2020). Beneficial effects of polysaccharide-rich extracts from *Apocynum venetum* leaves on hypoglycemic and gut microbiota in type 2 diabetic mice. Biomed. Rep..

[18] Shi L., Yili A., Aisa H., He F., Li Y.Q., Huang J.F. (2015). Purification process of total polyphenols from *Apocynum Venetum* L. leaves and its antioxidant activity. Her. Med..

[19] Xin X.L., Peng J., Liu X.Y., He F., Aisa H.A. (2019). Application of *Apocynum venetum* Leaf Polyphenol. ZL.

[20] Aisa H.A., Shi L.j., Yili A., He F. (2014). *Apocynum venetum* Leaf Total Polyphenol Preparation Method and Use. ZL.

[21] Ma Y., Li J., Tong F., Xin X.L., Aisa H.A. (2020). Optimization of microwave-assisted extraction using response surface methodology and the potential anti-diabetic efficacy of *Nigella glandulifera* Freyn determined using the spectrum-effect relationship. Ind. Crop. Prod..

[22] Liu X.Y., Peng J., He F., Tursun X., Li S.P., Xin X.L., Aisa H.A. (2022). Shabyar ameliorates high glucose iInduced retinal pigment epithelium injury through suppressing aldose reductase and AMPK/mTOR/ULK1 autophagy pathway. Front. Pharmacol..

[23] Arumugam B., Palanisamy U.D., Chua K.H., Kuppusamy U.R. (2020). Amelioration of hyperglycemia-induced oxidative damage in ARPE-19 cells by myricetin derivatives isolated from *Syzygium malaccense*. J. Funct. Foods.

[24] Milbury P.E., Graf B., Curran-Celentano J.M., Blumberg J.B. (2007). Bilberry (*Vaccinium myrtillus*) anthocyanins modulate heme oxygenase-1 and glutathione S-transferase-pi expression in ARPE-19 cells. Invest. Ophth. Vis. Sci..

[25] Senthilkumari S., Sharmila R., Chidambaranathan G., Vanniarajan A. (2017). Epalrestat, an aldose reductase Inhibitor prevents glucose-induced toxicity in human retinal pigment epithelial cells in vitro. J. Ocul. Pharmacol. Therapeut..

[26] Liu L., Yasen M., Tang D., Ye J., Aisa H.A., Xin X. (2018). Polyphenol-enriched extract of *Rosa rugosa* Thunb regulates lipid metabolism in diabetic rats by activation of AMPK pathway. Biomed. Pharmacother..

[27] Cao H., Chen X.Q. (2012). Structures required of flavonoids for inhibiting digestive enzymes. Anti-Cancer Agent. Med. Chem..

[28] Jung H.A., Park J.J., Islam M.N., Jin S.E., Min B.S., Lee J.H., Sohn H.S., Choi J.S. (2012). Inhibitory activity of coumarins from *Artemisia capillaris* against advanced glycation endproduct formation. Arch. Pharm. Res..

[29] Tang J., Du Y., Petrash J.M., Sheibani N., Kern T.S. (2013). Deletion of aldose reductase from mice inhibits diabetes-induced retinal capillary degeneration and superoxide generation. PLoS ONE.

[30] Xu Z., Sun T., Li W., Sun X. (2017). Inhibiting effects of dietary polyphenols on chronic eye diseases. J. Funct. Foods.

[31] Behl T., Kumar K., Singh S., Sehgal A., Sachdeva M., Bhatia S., Al-Harrasi A., Buhas C., Teodora Judea-Pusta C., Negrut N. (2021). Unveiling the role of polyphenols in diabetic retinopathy. J. Funct. Foods.

[32] Mathebula S.D. (2015). Polyol pathway: A possible mechanism of diabetes complications in the eye. Afr. Vis. Eye Health.

[33] Li X., Shen Y., Lu Y., Yang J. (2015). Amelioration of bleomycin-induced pulmonary fibrosis of rats by an aldose reductase inhibitor, epalrestat. Korean J. Physiol. Pharmacol..

[34] Xiao J., Ni X., Kai G., Chen X. (2015). Advance in dietary polyphenols as aldose reductases inhibitors: Structure-activity relationship aspect. Crit. Rev. Food Sci. Nutr..

[35] Chan A.W., Ho Y.S., Chung S.K., Chung S.S. (2008). Synergistic effect of osmotic and oxidative stress in slow-developing cataract formation. Exp. Eye Res..

[36] Yao L., Zhang N., Wang C., Wang C. (2015). Highly selective separation and purification of anthocyanins from Bilberry based on a macroporous polymeric adsorbent. J. Agric. Food Chem..

[37] Silva K.C., Rosales M.A.B., Hamassaki D.E., Saito K.C., Faria A.M., Ribeiro P.A.O., de Faria J.B.L., de Faria J.M.L. (2013). Green teais neuroprotective in diabetic retinopathy. Invest Ophth. Vis. Sci..

[38] Liu W.Y., Liou S.S., Hong T.Y., Liu I.M. (2017). The benefits of the Citrus flavonoid diosmin on human retinal pigment epithelial cells under high-glucose conditions. Molecules.

[39] Zhang Q., Li H.S., Li R., Du J.H., Jiao C. (2021). Autophagy dysregulation mediates the damage of high glucose to retinal pigment epithelium cells. Int. J. Ophthalmol..

[40] Huang C., Lu H., Xu J., Yu H., Wang X., Zhang X. (2018). Protective roles of autophagy in retinal pigment epithelium under high glucose condition via regulating PINK1/Parkin pathway and BNIP3L. Biol. Res..

[41] Szatmari-Toth M., Kristof E., Vereb Z., Akhtar S., Facsko A., Fesus L., Kauppinen A., Kaarniranta K., Petrovski G. (2016). Clearance of autophagy-associated dying retinal pigment epithelial cells—A possible source for inflammation in age-related macular degeneration. Cell Death Dis..

[42] Gao Y.Y., Li J., Huang J., Li W.J., Yu Y. (2022). Effects of Lycium barbarum polysaccharide on the photoinduced autophagy of retinal pigment epithelium cells. Int. J. Ophthalmol..

[43] Mauthe M., Orhon I., Rocchi C., Zhou X., Luhr M., Hijlkema K.J., Coppes R.P., Engedal N., Mari M., Reggiori F. (2018). Chloroquine inhibits autophagic flux by decreasing autophagosome-lysosome fusion. Autophagy.

[44] Chehri A., Yarani R., Yousefi Z., Shakouri S.K., Ostadrahimi A., Mobasseri M., Araj-Khodaei M. (2022). Phytochemical and pharmacological anti-diabetic properties of bilberries (*Vaccinium myrtillus*), recommendations for future studies. Prim. Care Diabetes.

[45] Wirth M., Joachim J., Tooze S.A. (2013). Autophagosome formation--the role of ULK1 and Beclin1-PI3KC3 complexes in setting the stage. Semin. Cancer Biol..

[46] Qiu X., Liu K., Xiao L., Jin S., Dong J., Teng X., Guo Q., Chen Y., Wu Y. (2018). Alpha-lipoic acid regulates the autophagy of vascular smooth muscle cells in diabetes by elevating hydrogen sulfide level. BBA-Mol. Basis Dis..

[47] Liu J., Tang Y., Feng Z., Liu J., Liu J., Long J. (2014). (−)-Epigallocatechin-3-gallate attenuated myocardial mitochondrial dysfunction and autophagy in diabetic Goto-Kakizaki rats. Free Radic. Res..

[48] Cheung N., Mitchell P., Wong T.Y. (2010). Diabetic retinopathy. Lancet.

[49] Pal P.B., Sonowal H., Shukla K., Srivastava S.K., Ramana K.V. (2019). Aldose reductase regulates hyperglycemia-induced HUVEC death via SIRT1/AMPK-alpha1/mTOR pathway. J. Mol. Endocrinol..

[50] Shukla K., Sonowal H., Saxena A., Ramana K.V., Srivastava S.K. (2017). Aldose reductase inhibitor, fidarestat regulates mitochondrial biogenesis via Nrf2/HO-1/AMPK pathway in colon cancer cells. Cancer Lett..

[51] Zhong K., Huang Y., Zilundu P.L.M., Wang Y., Zhou Y., Yu G., Fu R., Chung S.K., Tang Y., Cheng X. (2022). Motor neuron survival is associated with reduced neuroinflammation and increased autophagy after brachial plexus avulsion injury in aldose reductase-deficient mice. J. Neuroinflamm..

