# Supplementary material for: Polyphenol-Rich Extract of Apocynum venetum L. Leaves Protects Human Retinal Pigment Epithelial Cells against High Glucose-Induced Damage through Polyol Pathway and Autophagy
Source: Nutrients. 2024 Sep 2;16(17):2944. doi: 10.3390/nu16172944 (PMC11397065; doi:10.3390/nu16172944)
Supplement: Supplementary file 1 [file nutrients-16-02944-s001.zip › nutrients-3119325-supplementary.pdf]

Polyphenol-Rich Extract of *Apocynum venetum* L. Leaves Protects Human Retinal Pigment Epithelial Cells against High Glucose-Induced Damage through Polyol Pathway and Autophagy

Jun Peng [1,2], Rahima Abdulla [1], Xiaoyan Liu [1,2], Fei He [1], Xuelei Xin [1,*] and Haji Akber Aisa [1,*]

1 The State Key Laboratory Basis Xinjiang Indigenous Medicinal Plant Resource, Xinjiang Technical Institute of Physics and Chemistry, Chinese Academy of Sciences, Urumqi 830011, China; pengjunlhs@163.com (J.P.); rahima@ms.xjb.ac.cn (R.A.); liuxiaoyan_shawn@163.com (X.L.); hefei@ms.xjb.ac.cn (F.H.)

2 University of Chinese Academy of Sciences, Beijing 100039, China

* Correspondence: xinxl@ms.xjb.ac.cn (X.X.); haji@ms.xjb.ac.cn (H.A.A.)

Supplementary material

Figure S1 Cytotoxicity of AVL to ARPE-19 cells for 24 h in high glucose (30 mM) (A). Verification of high glucose damage model on the relative expression of the AR gene by quantitative real-time PCR (B), sorbitol content (C), and Na^+ K^+-ATPase activity (D). Data are analyzed using one- way ANOVA, and presented as the mean ± SD of three independent experiments. #P < 0.05, ##P < 0.01 versus NG, *P < 0.05, **P < 0.01 versus HG.

Figure S2 Determination of AVL concentration. The high glucose model was treated with different concentration of AVL (6.25, 12.5, 25 and 50 μg/mL) on sorbitol content (A) and Na^+ K^+ - ATPase activity (B). The high glucose model was treated with different time on AVL concentration of 25 μg/mL on sorbitol content (C) and Na^+ K^+ - ATPase activity (D). Data are analyzed using one- way ANOVA, and presented as the mean ± SD of three independent experiments. #P < 0.05, ##P < 0.01 versus NG, *P < 0.05, **P < 0.01 versus HG.

Figure S1

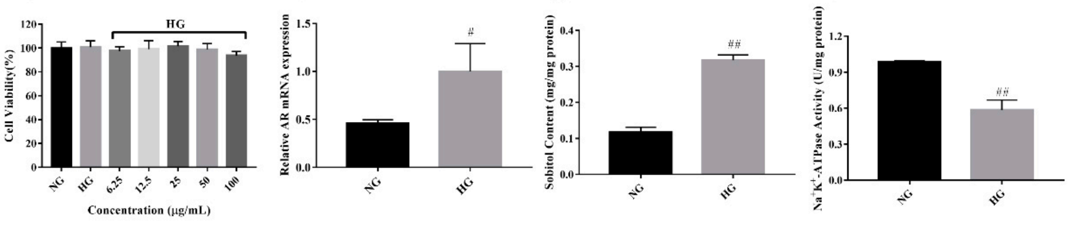

Figure S2

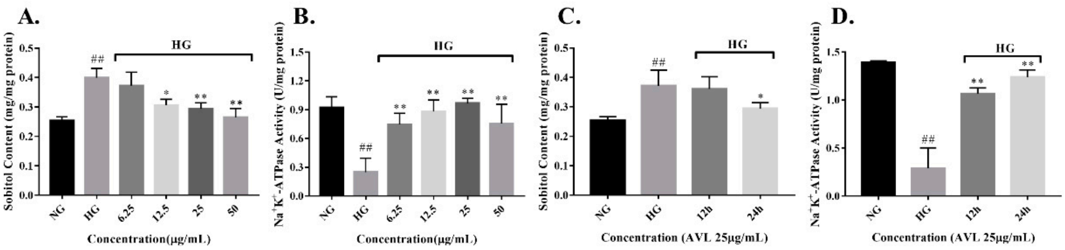

A.
Sobitol Content (mg/mg protein)
HG
Concentration(μg/mL)
B.
Na⁺K⁺-ATPase Activity (U/mg protein)
HG
Concentration(μg/mL)
C.
Sobitol Content (mg/mg protein)
HG
Concentration (AVL 25μg/mL)
D.
Na⁺K⁺-ATPase Activity (U/mg protein)
HG
Concentration (AVL 25μg/mL)
